# The roles of *Arabidopsis HSFA2*, *HSFA4a*, and *HSFA7a* in the heat shock response and cytosolic protein response

**DOI:** 10.1186/s40529-018-0231-0

**Published:** 2018-05-21

**Authors:** Kuan-Fu Lin, Meng-Yu Tsai, Chung-An Lu, Shaw-Jye Wu, Ching-Hui Yeh

**Affiliations:** Department of Life Sciences, National Central University, Taoyuan, 32001 Taiwan

**Keywords:** Azetidine-2-carboxylic acid, Cytosolic protein response, Heat shock factor, Heat shock protein, Heat shock response, Unfolded protein response

## Abstract

Previously, we found that *Arabidopsis* plants transformed with a construct containing the promoter of *Oshsp17.3* from rice fused to the β-glucuronidase gene (*GUS*), *Oshsp17.3Pro::GUS* (*Oshsp17.3p*), showed a *GUS* signal after heat shock (HS) or azetidine-2-carboxylic acid (AZC) treatment. HS and AZC trigger the heat shock response (HSR) and cytosolic protein response (CPR), respectively, in the cytosol by modulating specific heat shock factor (HSF) activity. Here we further identified that *AtHSFA2* (*At2g26150*), *AtHSFA7a* (*At3g51910*), *AtHSFB2a* (*At5g62020*), and *AtHSFB2b* (*At4g11660*) are HS- and AZC-inducible; *AtHSFA4a* (*At4g18880*) is AZC-inducible; and *AtHSFA5* (*At4g13980*) is less AZC- and HS-inducible. To investigate the roles of these 6 *AtHSFs* in the HSR or CPR, we crossed two independent *Oshsp17.3p* transgenic *Arabidopsis* plants with the *AtHSF*-knockout mutants *athsfa2* (SALK_008978), *athsfa4a* (GABI_181H12), *athsfa5* (SALK_004385), *athsfa7a* (SALK_080138), *athsfb2a* (SALK_137766), and *athsfb2b* (SALK_047291), respectively. As compared with the wild type, loss-of-function mutation of *AtHSFA2*, *AtHSFA4a*, and *AtHSFA7a* decreased HS and AZC responsiveness, so these 3 *AtHSFs* are essential for the HSR and CPR. In addition, loss-of-function results indicated that *AthsfB2b* is involved in regulating the HSR in *Arabidopsis*. Furthermore, analysis of the relative GUS activity of two double knockout mutants, *athsfA2*/*athsfA4a* and *athsfA2*/*athsfA7a*, revealed that *AtHSFA2*, *AtHSFA4a*, and *AtHSFA7a* function differentially in the HSR and CPR. Transcription profiling in *athsf* mutants revealed positive or negative transcriptional regulation among the 6 *AtHSFs* in *Arabidopsis* plants under HS and AZC conditions. Tunicamycin treatment demonstrated that these 6 *AtHSFs* are not involved in the unfolded protein response.

## Background

Protein homeostasis is crucial for maintaining normal cellular function. Plants, being sessile organisms, cannot escape from their growing environments. Extremes in environmental factors can result in stressful conditions that inevitably damage proteins directly or cause cells to synthesize misfolded proteins, which can lead to perturbed cell function and stress-induced cell death. Plants have evolved an extensive network of chaperone systems to restore protein folding or to remove irreversibly unfolded proteins (Mehdy 1994; Shinozaki and Yamaguchi-Shinozaki 1996; Bukau et al. 2006; Cramer et al. 2011; Redondo-Gómez 2013).

Accumulation of unfolded proteins within cells, eliciting compartment-specific chaperones and pathways, is termed the unfolded protein response (UPR). The UPR initiates the dissociation of the endoplasmic reticulum (ER) chaperone, immunoglobulin binding protein, and ER master sensors, such as inositol-requiring 1 and protein kinase R-like ER kinase, to activate downstream effectors to restore protein homeostasis in the lumen of the ER. A cytosolic process, the cytoplasmic protein response (CPR), increases the synthesis of molecular chaperones such as heat shock proteins (HSPs). In contrast to the better-understood UPR of the ER, the regulatory molecules in the CPR are not well elucidated.

The heat-shock response (HSR), predominantly a response to maintain protein-folding homeostasis in the cytosol, causes transcriptional activation of HSPs under thermal stress (Aparicio et al. 2005; Jungkunz et al. 2011). The expression of *HSP* genes is mainly under the control of heat shock transcription factors (HSFs) (Schöffl et al. 1998; Nover et al. 2001). The number of HSFs is characteristically higher in plants than in other organisms. For example, *Arabidopsis* and rice have 21 and 25 HSFs, respectively, but *Drosophila*, *C. elegans* and yeast have only one HSF (Nover et al. 2001; Guo et al. 2008; Scharf et al. 2012). The multiplicity of members of the HSF family in plants may contribute to their fitness to face varied environmental challenges such as extreme temperatures, drought, and salinity (Busch et al. 2005).

Plant HSFs are classified into three classes (A, B, and C) on the basis of structural characteristics and phylogenetic comparison. Class A HSFs contain a DNA binding domain, an oligomerization domain, nuclear localization domains, and transcriptional activation domains. Classes B and C lack a transcriptional activation domain (Nover et al. 2001). Recent studies of tomato *HSFA1a* mutants and an *Arabidopsis HSFA1a/1b/1d/1e* quadruple mutant revealed that members of *HSFA1* genes can function as master regulators for the HSR and play important roles in cross-regulation for abiotic stress responses (Mishra et al. 2002; Liu et al. 2011). Increasing evidence shows functional diversification among different HSF members.

In addition to heat shock (HS), a proline analog, azetidine-2-carboxylic acid (AZC), can induce accumulation of abnormal-misfolded proteins in the cytosol to trigger the CPR by modulating HSFA2 activity (Yeh et al. 2007; Sugio et al. 2009; Nishizawa-Yokoi et al. 2011). In the current study, we fused the promoter of AZC-inducible small *HSP* (*sHSP*), *Oshsp17.3*, with the *β*-*glucuronidase* gene (*GUS*) (*Oshsp17.3Pro::GUS*) and transformed into *Arabidopsis AtHSF* mutants, and detected GUS activity in response to AZC and HS (Guan et al. 2010). Our results allowed us to characterize the roles of *Arabidopsis HSFs* in the HSR and CPR.

## Methods

### Plant materials

The *Arabidopsis thaliana* ecotype *Col*-*0* was used in this study as the wild type (WT). Seeds were surface-sterilized in commercial bleach that contained 5% (v/v) sodium hypochlorite and 0.1% (v/v) Triton X-100 solution for 10 min, rinsed in sterilized water, and stratified at 4 °C for 2 days in the dark. Seeds were germinated on growth agar plates [1/2 Murashige and Skoog medium (MS; Duchefa), 1% sucrose (w/v), 0.8% agar (w/v)]. The T-DNA insertion lines SALK_008978 (*athsfa2*), GABI_181H12 (*athsfa4a*), SALK_004385 (*athsfa5*), SALK_080138 (*athsfa7a*), SALK_137766 (*athsfb2a*), and SALK_047291 (*athsfb2b*) mutants were obtained from the Arabidopsis Biological Resources Center (ABRC, Columbus, OH, USA) (Liu et al. 2011; Kleinboelting et al. 2012). The *athsfa2/athsfa4a* and the *athsfa2/athsfa7a* double mutants were generated by crossing *athsfa2* with *athsfa4a* and *athsfa7a* mutants. Mutant seeds were germinated and selected on selection agar plates [1/2 MS, 1% sucrose (w/v), 25 μg/ml hygromycin, 0.8% agar (w/v)]. All seedlings were grown at 23 °C in a 16-h light/8-h dark cycle in a growth chamber with 60% relative humidity.

### RNA isolation and RT-PCR

Total RNA was extracted from 10-day-old *Arabidopsis* seedlings as described (Guan et al. 2010). The first-strand cDNA was synthesized with 1 μg total RNA by using the SuperScript III First-Stand Synthesis System (Invitrogen). PCR amplification corresponding to different *AtHSFs* shown in Fig. 3 were 30 s at 94 °C, 30 s at 52 °C, and 30 s at 72 °C, then 5 min at 72 °C. Primers used for analysis of gene expression were designed by use of NCBI Primer-BLAST (https://www.ncbi.nlm.nih.gov/tools/primer-blast/) and are in Table 1. DNA from 15 μl of each PCR reaction was fractionated by electrophoresis on 1.2% (w/v) agarose gel with 0.01% (w/v) ethidium bromide in 1× Tris–Acetate EDTA buffer. The gel was digitally photographed and the corresponding DNA signal was quantified by using ImageJ (http://rsbweb.nih.gov/ij/) (Schneider et al. 2012) and normalized to *18S* rRNA expression.

**Table 1:** Oligonucleotides used in RT-PCR

Gene	Primer name	Sequence
*AtHSFA2*	AtHSFA2-Fw	5′-CCATGGAAGAACTGAAAGTGGAAATGGAGG-3′
	AtHSFA2-Rv	5′-GCGGCCGCAGGTTCCGAACCAAG-3′
*AtHSFA4a*	AtHSFA4a-Fw	5′-CATCAAGTGGAACAGTTAGA-3′
	AtHSFA4a-Rv	5′-ACTCCGGCTTTATCTTTATC-3′
*AtHSFA5*	AtHSFA5-Fw	5′-AGCAAGAGTGAATGATGTAT-3′
	AtHSFA5-Rv	5′-CTACTTACGCTTTTTCAGTC-3′
*AtHSFA7a*	AtHSFA7a-Fw	5′-ATCAAAGCTATGGAACAGAG-3′
	AtHSFA7a-Rv	5′-AACTCTCATCACTAAGCAAC-3′
*AtHSFB2a*	AtHSFB2a-Fw	5′-TTGAGACATTATAATCGAAC-3′
	AtHSFB2a-Rv	5′-TCTAAAAATGTACTTGTGAT-3′
*AtHSFB2b*	AtHSFB2b Fw	5′-GAGGAGAATAACTCCGGTAA-3′
	AtHSFB2b Rv	5′-ATGCAATGGGGATCAGTAAC-3′
*AtTubulin*	AtTubulin Fw	5′-GCCAATCCGGTGCTGGTAACA-3′
	AtTubulin Rv	5′-CATACCAGATCCAGTTCCTCCTCCC-3′
*AtbZIP60*	AtbZIP60-Fw	5′-AGGACGTATGCTTGAGTGCTTCGT-3′
	AtbZIP60-Rv	5′-TTCTGGACGTAGGAGGCAACACT-3′
*GUS*	GUS-Fw	5′-GGCCTGTGGGCATTCAGTCT-3′
	GUS-Rv	5′-AGTTCAGTTCGTTGTTCACACAA-3′

### Preparation of DNA constructs and transformation

*Oshsp17.3Pro::GUS* (*Oshsp17.3p*) and *Oshsp17.3Pro*-*ΔAZRE::GUS* (*Oshsp17.3pΔAZRE*) were constructed and transformed *Arabidopsis* plants as described (Guan et al. 2010). Transgenic plants #5 and #11 of *Oshsp17.3Pro::GUS* (*Oshsp17.3p5* and *Oshsp17.3p11*, respectively), which showed *GUS* expression induced by HS and AZC (Guan et al. 2010), were selected to cross with *AtHSF* mutants *athsfA2*, *athsfA4a*, *athsfA5*, *athsfA7a*, *athsfB2a*, *athsfB2b*, *athsfA2/athsfA4a*, and *athsfA2/athsfA7a* mutants, respectively. F2 lines *Oshsp17.3p5/athsfA2*, *Oshsp17.3p5/athsfA4a*, *Oshsp17.3p5/athsfA5*, *Oshsp17.3p5/athsfA7a*, *Oshsp17.3p5/athsfB2a*, *Oshsp17.3p5/athsfB2b*, *Oshsp17.3p5/athsfA2/athsfA4a*, *Oshsp17.3p5/athsfA2/athsfA7a*, *Oshsp17.3p11/athsfA2*, *Oshsp17.3p11/athsfA4a*, *Oshsp17.3p11/athsfA5*, *Oshsp17.3p11/athsfA7a*, *Oshsp17.3p11/athsfB2a*, *Oshsp17.3p11/athsfB2b*, *Oshsp17.3p11/athsfA2/athsfA4a*, and *Oshsp17.3p11/athsfA2/athsfA7a* were obtained and then self-pollinated to produce the F3 generation, which was used for analysis of HS and AZC responsiveness in this study. In addition, transgenic plants #2 and #7 of *Oshsp17.3Pro*-*ΔAZRE::GUS* (*Oshsp17.3pΔAZRE*), which showed weak *GUS* expression with HS and AZC treatment (Guan et al. 2010), were used as the negative control.

### Stress treatment of transgenic *Arabidopsis* mutants

For HS treatment, 10-day-old F3-generation *Arabidopsis* seedlings were incubated in shaking buffer [1% sucrose (w/v), 5 mM potassium phosphate buffer, pH 6.8] at 39 °C for 1 h, then 23 °C for 20 h of recovery. For AZC treatment, 10-day-old F3-generation *Arabidopsis* seedlings were incubated in shaking buffer with or without 5 mM AZC at 23 °C for 4 h, rinsed in sterilized water, then incubated in shaking buffer at 23 °C for 15 h of recovery. For tunicamycin (Tm) treatment, 10-day-old F3-generation *Arabidopsis* seedlings were incubated in shaking buffer with or without 5 μg/ml Tm at 23 °C for 4 h, rinsed in sterilized water, then incubated in shaking buffer at 23 °C for 15 h of recovery. All samples were frozen by liquid nitrogen and stored at − 80 °C.

### GUS staining

GUS staining was described previously (Guan et al. 2010). In brief, 10-day-old seedlings were treated and incubated in the fixation solution (0.3% formaldehyde, 0.1% Triton X-100, 0.1% β-mercaptoethanol, 100 mM sodium phosphate buffer, pH 7.0) for 60 min. Then the fixation solution was replaced with washing solution (100 mM sodium phosphate buffer, 1 mM EDTA, pH 7.0) twice for 15 min. Washed seedlings were vacuum-infiltrated for 5 min in GUS staining buffer (1 mM X-Gluc, 0.5 mM ferricyanide, 0.5 mM ferrocyanide, 0.1% Triton X-100, 10 mM EDTA, 100 mM sodium phosphate buffer, pH 7.0), then incubated at 37 °C for 24 h. The staining reaction was stopped by adding distilled water, the color of chlorophyll was removed with 70% ethanol (v/v) several times, and seedlings were soaked in 95% ethanol (v/v) for 1 h. Plants were photographed to record deposition of the GUS.

### Analysis of GUS activity

Seedlings after HS or AZC treatment were powdered in liquid nitrogen and extracted with GUS extraction buffer (50 mM sodium phosphate buffer, 10 mM EDTA, 0.1% SDS, 0.1% triton X-100, 0.1% β-mercaptoethanol, 1 mM PMSF, pH 7.0). After centrifugation, 10-μl protein extract was mixed with 990-μl GUS assay solution [2.5 mM MUG, 50 mM NaPO4, 10 mM EDTA, 10 mM DTT, 2% Leupeptin (w/v), 20% methanol (v/v), pH 7.0], which was preheated in 37 °C for 5 min, and extract was incubated in 37 °C for 1 h. For GUS activity assay, the fluorescence was measured in a Fluoroskan Ascent FL fluorometer (Labsystems, Helsinki, Finland).

### Statistical analysis

Data are shown as mean ± SE from three independent experiments. Statistical differences were analyzed by Student *t* test or Duncan multiple range test. *P *< 0.05 was considered statistically significant.

## Results

### Transcript levels of *AtHSFs* under heat and AZC stress

**Fig. 1:**
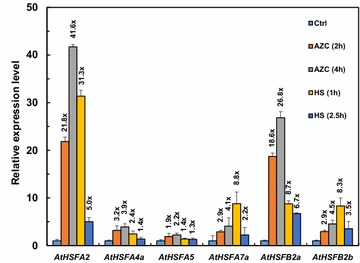
Expression profiles of *AtHSFs* in *Arabidopsis* plants under AZC and HS treatment. RT-PCR analysis of transcript levels in wild-type (WT) seedlings treated with AZC and HS for the indicated times. Data are mean ± SE expression relative to that of non-stressed control (Ctrl) from three independent experiments and the fold expression is indicated

*HSFA2*, *HSFA7a*, *HSFB1*, *HSFB2a*, and *HSFB2b* were previously found as AZC- and HS-inducible *HSFs* in *Arabidopsis* seedlings (Sugio et al. 2009). To further confirm the responsiveness of *Arabidopsis HSFs* to AZC and HS under our test conditions, we analyzed transcript levels of *Arabidopsis HSFs* under AZC and heat treatments (data not shown). We selected highly AZC- and HS-inducible *AtHSFA2* (*At2g26150*; 41.8–21.8-fold and 31.3–5-fold induction, respectively), *AtHSFA7a* (*At3g51910*; 4.1–2.9-fold and 8.8–2.2-fold, respectively), *AtHSFB2a* (*At5g62020*; 26.8–18.6-fold and 8.7–6.7-fold, respectively), and *AtHSFB2b* (*At4g11660*; 4.5–2.9-fold and 8.3–3.5-fold, respectively) as candidate *HSFs* for further study (Fig. 1). In addition, *AtHSFA4a* (*At4g18880*), which showed AZC responsiveness (3.9–3.2-fold), and *AtHSFA5* (*At4g13980*), which showed less AZC and HS responsiveness, were included in the test.

### *AtHSFA2*, *AtHSFA4a*, and *AtHSFA7a* genes function differentially in response to heat and AZC stress

To investigate whether the *AtHSFs* examined are involved in the HS or AZC responsiveness, Two independent *Oshsp17.3Pro::GUS* transgenic plants, *Oshsp17.3p5* and *Oshsp17.3p11*, were separately crossed with *athsfA2*, *athsfA4a*, *athsfA5*, *athsfA7a*, *athsfB2a*, and *athsfB2b* mutants. Lines *Oshsp17.3p5/athsfA2*, *Oshsp17.3p5/athsfA4a*, *Oshsp17.3p5/athsfA5*, *Oshsp17.3p5/athsfA7a*, *Oshsp17.3p5/athsfB2a*, *Oshsp17.3p5/athsfB2b*, *Oshsp17.3p5/athsfA2/athsfA4a*, *Oshsp17.3p5/athsfA2/athsfA7a*, *Oshsp17.3p11/athsfA2*, *Oshsp17.3p11/athsfA4a*, *Oshsp17.3p11/athsfA5*, *Oshsp17.3p11/athsfA7a*, *Oshsp17.3p11/athsfB2a*, *Oshsp17.3p11/athsfB2b*, *Oshsp17.3p11/athsfA2/athsfA4a*, and *Oshsp17.3p11/athsfA2/athsfA7a* were obtained for analyzing HS and AZC responsiveness.

**Fig. 2:**
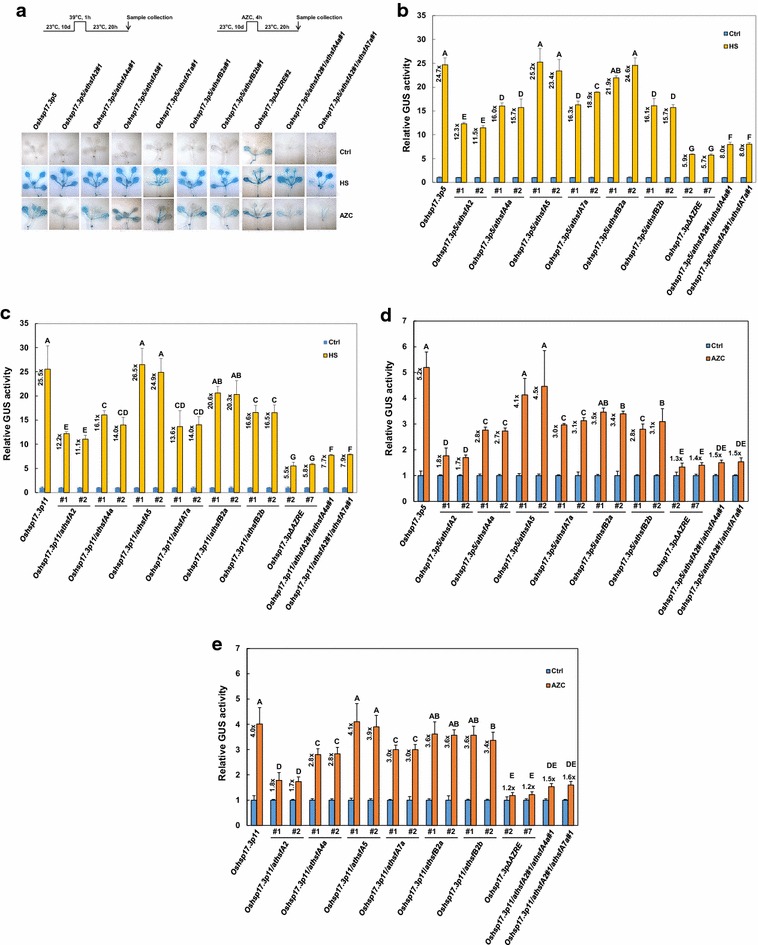
Analysis of HS and AZC responsiveness of *AtHSF* mutants by GUS staining. *AtHSF* mutant plants were transformed with a chimeric *Oshsp17.3Pro::GUS* gene as described. **a** Seedlings from independent transgenic lines underwent HS or AZC treatment as indicated and GUS histochemical staining. Non-stress control condition (Ctrl). Relative GUS activity of seedlings treated with **b**, **c** HS and **d**, **e** AZC. Data are mean ± SE GUS activity relative to that of the Ctrl from three independent experiments and the fold expression is indicated. Bars with the same letter are not significantly different at *P *< 0.05

Under the HS condition (39 °C for 1 h), *Oshsp17.3p5* plants showed GUS staining; *Oshsp17.3p5/athsfA2*, *Oshsp17.3p5/athsfA4a*, *Oshsp17.3p5/athsfA7a*, and *Oshsp17.3p5/athsfB2b* plants showed reduced GUS expression; and GUS staining was similar in *Oshsp17.3p5/athsfA5* and *Oshsp17.3p5/athsfB2a* plants (Fig. 2a). Under AZC treatment (5 mM AZC for 4 h), both cotyledons and true leaves of *Oshsp17.3p5/athsfA2* and *Oshsp17.3p5/athsfA7a* plants did not show any GUS signal (Fig. 2a), and true leaves of *Oshsp17.3p5/athsfA4a*, *Oshsp17.3p5/athsfB2a*, and *Oshsp17.3p5/athsfB2b* plants showed little or no GUS signal; the profile of GUS staining was similar in *Oshsp17.3p5* and *Oshsp17.3p5/athsfA5* plants. Similar HS- and AZC-induced profile of GUS staining was found in *Oshsp17.3p11*, *Oshsp17.3p11/athsfA2*, *Oshsp17.3p11/athsfA4a*, *Oshsp17.3p11/athsfA5*, *Oshsp17.3p11/athsfA7a*, *Oshsp17.3p11/athsfB2a*, *Oshsp17.3p11/athsfB2b*, *Oshsp17.3p11/athsfA2/athsfA4a*, and *Oshsp17.3p11/athsfA2/athsfA7a* (data not shown).

The reduction in HS and AZC responsiveness measured by GUS activity was further confirmed quantitatively. With HS treatment, GUS activity was about 55% lower for *Oshsp17.3p5/athsfA2* than *Oshsp17.3p5* plants (Fig. 2b). Also, GUS activity was lower for *Oshsp17.3p5/athsfA4a*, *Oshsp17.3p5/athsfA7a*, and *Oshsp17.3p5/athsfB2b* than *Oshsp17.3p5* plants (36, 24–34, and 36% reduction, respectively). Similar reduction of GUS activity was further confirmed in *Oshsp17.3p11/athsfA2*, *Oshsp17.3p11/athsfA4a*, *Oshsp17.3p11/athsfA7a*, and *Oshsp17.3p11/athsfB2b* compared with *Oshsp17.3p11* plants (Fig. 2c). We did not find a significant difference in GUS activity among *Oshsp17.3p5*, *Oshsp17.3p11*, *Oshsp17.3p5/athsfA5*, *Oshsp17.3p11/athsfA5*, *Oshsp17.3p5/athsfB2a* and *Oshsp17.3p11/athsfB2a* plants. These loss-of-function results indicate that mutation of *AthsfA2*, *AthsfA4a*, *AthsfA7a*, and *AthsfB2b* may alter HS responsiveness in *Arabidopsis* plants.

We then compared the effect of *AtHSF* mutation on AZC responsiveness. With AZC treatment, relative GUS activity was lower for *Oshsp17.3p5/athsfA2*, *Oshsp17.3p5/athsfA4a*, and *Oshsp17.3p5/athsfA7a* than *Oshsp17.3p5* plants (65–67, 46–48, and 40–42% reduction, respectively) (Fig. 2d) but did not significantly differ among *Oshsp17.3p5*, *Oshsp17.3p5/athsfA5*, *Oshsp17.3p5/athsfB2a*, and *Oshsp17.3p5/athsfB2b* plants. Similar reduction of GUS activity was further confirmed in *Oshsp17.3p11/athsfA2*, *Oshsp17.3p11/athsfA4a*, and *Oshsp17.3p11/athsfA7a* compared with *Oshsp17.3p11* plants (Fig. 2e). Thus, on GUS activity analysis of HS- and AZC-treated seedlings, *AtHSFA2*, *AtHSFA4a*, and *AtHSFA7a* were important for the HSR and AZC response in *Arabidopsis*.

Furthermore, we crossed *athsfA2*/*athsfA4a* and *athsfA2*/*athsfA7a* plants with *OsHsp17.3p5* and *OsHsp17.3p11* transgenic *Arabidopsis*, respectively and obtained *Oshsp17.3p5/athsfA2*/*athsfA4a*, *Oshsp17.3p11/athsfA2*/*athsfA4a*, *Oshsp17.3p5/athsfA2*/*athsfA7a*, and *Oshsp17.3p11/athsfA2*/*athsfA7a Arabidopsis* plants for testing HS and AZC responsiveness. With HS treatment, GUS signal was absent in true leaves of *Oshsp17.3p5/athsfA2*/*athsfA4a* and cotyledons of *Oshsp17.3p5/athsfA2*/*athsfA7a*, and AZC-induced GUS signal was not significant in *Oshsp17.3p5/athsfA2*/*athsfA4a* or *Oshsp17.3p5/athsfA2*/*athsfA7a Arabidopsis* plants, which was similar to *Oshsp17.3p5/athsfA2* and *Oshsp17.3pΔAZRE* plants (Fig. 2a). Similar HS- and AZC-induced profile of GUS staining was found in *Oshsp17.3p11/athsfA2*/*athsfA4a* and *Oshsp17.3p11/athsfA2*/*athsfA7a Arabidopsis* plants (data not shown). On quantitative analysis, with HS treatment, relative GUS activity was significantly lower for *Oshsp17.3p5/athsfA2*/*athsfA4a* and *Oshsp17.3p5/athsfA2*/*athsfA7a* than *Oshsp17.3p5/athsfA2*, *Oshsp17.3p5/athsfA4a*, and *Oshsp17.3p5/athsfA7a* plants (Fig. 2b). Also, GUS activity was lower for *Oshsp17.3p11/athsfA2*/*athsfA4a* and *Oshsp17.3p11/athsfA2*/*athsfA7a* than *Oshsp17.3p11/athsfA2*, *Oshsp17.3p11/athsfA4a*, and *Oshsp17.3p11/athsfA7a* plants (Fig. 2c). With AZC treatment, the GUS activity for *Oshsp17.3p5/athsfA2*/*athsfA4a* and *Oshsp17.3p5/athsfA2*/*athsfA7a* plants dropped to a level (38 and 40% of GUS activity, respectively, versus *Oshsp17.3p5* plants) comparable to that for *Oshsp17.3p5/athsfA2* and *Oshsp17.3pΔAZRE* plants (Fig. 2d). In addition, GUS activity did not significantly differ among *Oshsp17.3p11/athsfA2*/*athsfA4a*, *Oshsp17.3p11/athsfA2*/*athsfA7a*, and *Oshsp17.3p11/athsfA2* plants (Fig. 2e). These results suggest that *AtHSFA2*, *AtHSFA4a*, and *AtHSFA7a* genes function independently in the HSR of *Arabidopsis* plants.

### Positive and negative regulation among the *AtHSF*s

**Fig. 3:**
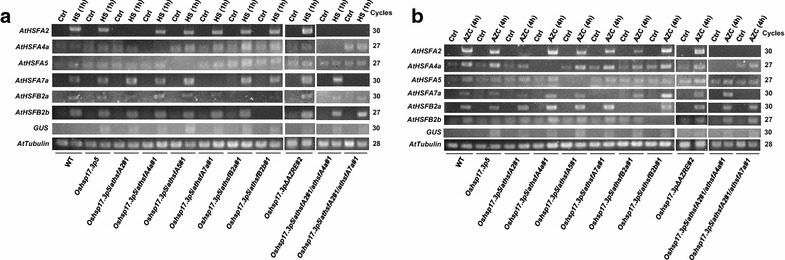
Expression profiles of *AtHSFs* in *AtHSF* mutants with HS and AZC treatment. RT-PCR analysis of transcript levels in the *AtHSF*-knockout mutant seedlings treated with **a** HS and **b** AZC for the indicated times. *AtTubulin* level was an internal control. Two biological repeats were performed, and similar results were obtained. Non-stress control condition (Ctrl)

Data in Fig. 1 revealed that *AtHSFA2*, *AtHSFA7a*, *AtHSFB2a*, and *AtHSFB2b* were HS- and AZC-inducible and *AtHSF4a* was AZC-inducible. We examined the expression profiles of the 6 *AtHSFs* in the mutants under stress. After 1 h of heat treatment, compared with the WT and *Oshsp17.3p5* plants, *AtHSFA4a* transcript level was significantly elevated in *Oshsp17.3p5/athsfA5* and *Oshsp17.3p5/athsfB2a* plants and *AtHSFA7a* level was increased in *Oshsp17.3p5/athsfA2*, *Oshsp17.3p5/athsfA5*, *Oshsp17.3p5/athsfB2a*, and *Oshsp17.3p5/athsfA2*/*athsfA4a* plants, with no significant change in *AtHSFA2*, *AtHSFA5*, *AtHSFB2a*, and *AtHSFB2b* levels in the mutant plants tested (Fig. 3a). With AZC treatment, *AtHSFA2* and *AthsfA4a* levels were reduced in *Oshsp17.3p5/athsfB2a* and *Oshsp17.3p5/athsfA2* plants, respectively, and *AtHSFA7a* level was increased in *Oshsp17.3p5/athsfA4a* and *Oshsp17.3p/athsfB2b* plants (Fig. 3b). Similar expression profiles of the 6 *AtHSFs* were also found in *Oshsp17.3p11*, *Oshsp17.3p11/athsfA2*, *Oshsp17.3p11/athsfA4a*, *Oshsp17.3p11/athsfA5*, *Oshsp17.3p11/athsfA7a*, *Oshsp17.3p11/athsfB2a*, *Oshsp17.3p11/athsfB2b*, *Oshsp17.3p11/athsfA2/athsfA4a*, and *Oshsp17.3p11/athsfA2/athsfA7a* plants under HS and AZC conditions (data not shown). These results suggest a finely tuned activation and repression of the expression of *HSFs* under HS and AZC stress.

### *AtHSFA2*, *AtHSFA4a*, and *AtHSFA7a* are not responsive to Tm

**Fig. 4:**
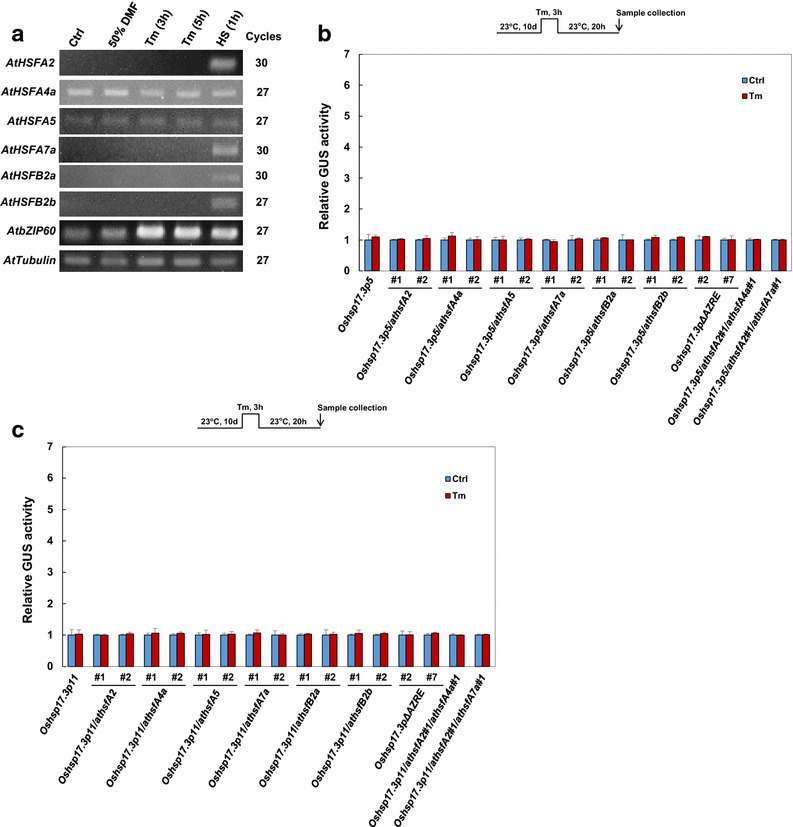
Analysis of tunicamycin (Tm) responsiveness of *AtHSF* mutants. **a** RT-PCR analysis of transcript levels in WT seedlings treated with Tm or solvent (dimethylformamide; DMF) and HS for the indicated times. mRNA expression of *AtbZIP60* was a positive control for HS and Tm treatment. *AtTubulin* level was an internal control. Two biological repeats were performed, and similar results were obtained. **b**, **c** Relative GUS activity of seedlings treated with Tm. Data are mean ± SE GUS activity relative to that of non-stress control condition (Ctrl) from three independent experiments

AZC typically induces the UPR and CPR. The data in Fig. 2 indicated that *AtHSFA2*, *AtHSFA4a*, and *AtHSFA7a* are essential for the HSR and AZC response in *Arabidopsis*. Studies have shown *AtHSFA2* as a crucial regulatory component of the CPR (Sugio et al. 2009). To understand whether these *AtHSFs* are involved in the UPR, we examined the effect of Tm treatment (UPR induction) in the *AtHSF* mutants tested. Tm did not activate the expression of the 6 *AtHSF* genes (Fig. 4a). On GUS analysis, no Tm responsiveness was detected in the mutant plants tested (Fig. 4b, c). These results confirmed that *AtHSFA2*, *AtHSFA4a*, and *AtHSFA7a* function in the CPR.

## Discussion

To adapt to biotic and abiotic stresses, plants have evolved a complex set of molecular responses, which often exhibit features sharing substantial overlap pathways and components. *HSF*/*HSP* responses are recognized as central chaperone components against unfolded protein accumulation, a signal for triggering HSR, UPR, or CPR based on distinct subcellular localization (Aparicio et al. 2005; Swindell et al. 2007; Yeh et al. 2007). Many reports have shown that HSFs are important for resistance to heat and other environmental stresses (Mishra et al. 2002; Charng et al. 2007; Banti et al. 2010; Liu et al. 2011). Using an HS- and AZC-sensitive promoter-*GUS* fusion system (Guan et al. 2010) together with knockout plants, we aimed to identify the contribution of *AtHSFA2*, *AtHSFA4a*, *AtHSFA5*, *AtHSFA7a*, *AtHSFB2a*, and *AtHSFB2b* to the responses induced by HS, AZC, and Tm.

Plant *HSFs* are regulated by HS and AZC, including up- and downregulation. We found the expression of *AtHSFA2*, *AtHSFA4a*, *AtHSFA7a*, *AtHSFB2a*, and *AtHSFB2b* induced > twofold with 1-h HS treatment and then reduced after prolonged heat incubation (Fig. 1). As well, AZC upregulated *AtHSFA2*, *AtHSFA4a*, *AtHSFA7a*, *AtHSFB2a*, and *AtHSFB2b* expression > 2.9-fold during treatment. However, Tm did not affect the expression of the 6 *AtHSFs* (Fig. 4a). Despite a slight difference in plant material and treatment time, the results are similar to published microarray data (Busch et al. 2005; Schramm et al. 2008; Sugio et al. 2009), finding that *AtHSFA2*, *AtHSFA4a*, *AtHSFA7a*, *AtHSFB2a*, and *AtHSFB2b* are important for stress response networks.

Studies have shown that *AtHSFA2* and *AtHSFA7a* knockout mutants lose acquired thermotolerance, and *AtHSFA2* mutants also show reduced tolerance to AZC (Charng et al. 2007; Siddique et al. 2008; Sugio et al. 2009). In this study, loss-of-function mutation of *AtHSFA2* significantly repressed relative GUS activity under HS and AZC treatment (Fig. 2b–e). By contrast, null mutation of *AtHSFA4a* and *AtHSFA7a* only slightly repressed relative GUS activity under HS and AZC stress. These results agree with others showing that *AtHSFA2* is closely related to the regulation of HSR as well as CPR (Busch et al. 2005; Nishizawa et al. 2006; Ogawa et al. 2007; Sugio et al. 2009; Jung et al. 2010), whereas *AtHSFA4a* and *AtHSFA7a* have a lesser effect on HSR and CPR. Furthermore, as compared with *AtHSFA2* knockout alone, double knockout with *AtHSFA2* and *AtHSFA4a* or *AtHSFA7a* showed more significant repression of HS-induced GUS activity (Fig. 2b–e). Thus, *AtHSFA2*, *AtHSFA4a*, and *AtHSFA7a* may be linked to activation of different target genes/pathways in the HSR. However, *AtHSFA2* appears to be a functionally redundant factor to *AtHSFA4a* and *AtHSFA7a* for AZC-induced CPR because the GUS activity of *AtHSFA2*-knockout plants was similar to that with double knockout of *AtHSFA2* and *AtHSFA4a* or *AtHSFA7a* under AZC treatment (Fig. 2b–e).

Ikeda et al. (2011) reported that *AtHsfB1* and *AtHsfB2b*, sharing functional redundancy in repressive activities, were able to suppress the accumulation of *AtHSFA2* and *AtHSFA7a* transcripts and were indispensable for acquired thermotolerance. As compared with *AtHSFA2* knockout, *AtHsfB2b* knockout slightly repressed GUS activity in response to HS treatment (Fig. 2b, c). We also revealed no significant change in HS-induced *AtHSFA2* and *AtHSFA7a* transcript levels with *AtHsfB2b* knockout (Fig. 3a). These results suggest that *AtHSFB2b* may mediate the HSR but not CPR. Of note, *AtHSFB2a* is highly AZC- and HS-inducible, but we did not find a significant reduction in GUS activity with *AtHsfB2a* knockout during AZC treatment. However, we cannot absolutely exclude the role of *AtHsfB2a* in AZC-induced CPR because of its high expression under AZC and HS treatment.

**Fig. 5:**
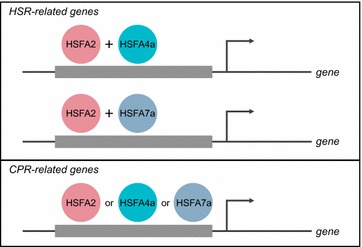
Working model of the AtHSFs involved in HSR and CPR. Gray bar indicates the promoter region of HSR- and CPR-related genes. Arrow indicates transcription starting site

In conclusion, we confirmed and characterized the roles of *AtHSFA2*, *AtHSFA4a*, *AtHSFA5*, *AtHSFA7a*, *AtHSFB2a*, and *AtHSFB2b* in the HSR and CPR. For simplifying our result, we propose a working model to show the roles of following AtHSFs in CPR and HSR (Fig. 5). *AtHSFA2*, *AtHSFA4a*, and *AtHSFA7a* function independently in the HSR, but *AtHSFA2* may function redundantly with *AtHSFA4a* and *AtHSFA7a* in the CPR. *AtHSFB2b* has some role in mediating the HSR, and *AtHSFA5 and AtHSFB2a* cannot mediate the HSR and CPR. These 6 *AtHSFs* are not involved in the UPR.

## Abbreviations

AZC: azetidine-2-carboxylic acid
AZRE: azetidine-2-carboxylic acid response element
CPR: cytosolic protein response
HSF: heat shock factor
HSP: heat shock protein
HSR: heat shock response
UPR: unfolded protein response
Tm: tunicamycin

## References

[CR1] Aparicio F, Aparicio F, Thomas CL, Lederer C, Niu Y, Wang D, Maule AJ (2005). Virus induction of heat shock protein 70 reflects a general response to protein accumulation in the plant cytosol. Plant Physiol.

[CR2] Banti V, Mafessoni F, Loreti E, Alpi A, Perata P (2010). The heat-inducible transcription factor *HsfA2* enhances anoxia tolerance in *Arabidopsis*. Plant Physiol.

[CR3] Bukau B, Weissman J, Horwich A (2006). Molecular chaperones and protein quality control. Cell.

[CR4] Busch W, Wunderlich M, Schöffl F (2005). Identification of novel heat shock factor-dependent genes and biochemical pathways in *Arabidopsis thaliana*. Plant J.

[CR5] Charng Y-Y, Liu H-C, Liu N-Y, Chi W-T, Wang C-N, Chang S-H, Wang T-T (2007). A heat-inducible transcription factor, HsfA2, is required for extension of acquired thermotolerance in *Arabidopsis*. Plant Physiol.

[CR6] Cramer GR, Urano K, Delrot S, Pezzotti M, Shinozaki K (2011). Effects of abiotic stress on plants: a systems biology perspective. BMC Plant Biol.

[CR7] Guan JC, Yeh CH, Lin YP, Ke YT, Chen MT, You JW, Liu YH, Lu CA, Wu SJ, Lin CY (2010). A 9 bp cis-element in the promoters of class I small heat shock protein genes on chromosome 3 in rice mediates l-azetidine-2-carboxylic acid and heat shock responses. J Exp Bot.

[CR8] Guo J, Wu J, Ji Q, Wang C, Luo L, Wang Y, Wang J (2008). Genome-wide analysis of heat shock transcription factor families in rice and *Arabidopsis*. J Genet Genomics.

[CR30] Ikeda M., Mitsuda N., Ohme-Takagi M. (2011). Arabidopsis HsfB1 and HsfB2b Act as Repressors of the Expression of Heat-Inducible Hsfs But Positively Regulate the Acquired Thermotolerance. Plant Physiol.

[CR9] Jung K-H, Cao P, Seo Y-S, Dardick C, Ronald PC (2010). The Rice Kinase Phylogenomics Database: a guide for systematic analysis of the rice kinase super-family. Trends Plant Sci.

[CR10] Jungkunz I, Link K, Vogel F, Voll LM, Sonnewald S, Sonnewald U (2011). AtHsp70-15-deficient *Arabidopsis* plants are characterized by reduced growth, a constitutive cytosolic protein response and enhanced resistance to TuMV. Plant J.

[CR11] Kleinboelting N, Huep G, Kloetgen A, Viehoever P, Weisshaar B (2012). GABI-Kat SimpleSearch: new features of the *Arabidopsis thaliana T*-*DNA* mutant database. Nucleic Acids Res.

[CR12] Liu HC, Liao HT, Charng YY (2011). The role of class A1 heat shock factors (*HSFA1s*) in response to heat and other stresses in *Arabidopsis*. Plant Cell Environ.

[CR13] Mehdy MC (1994). Active oxygen species in plant defense against pathogens. Plant Physiol.

[CR14] Mishra SK, Tripp J, Winkelhaus S, Tschiersch B, Theres K, Nover L, Scharf K-D (2002). In the complex family of heat stress transcription factors, *HsfA1* has a unique role as master regulator of thermotolerance in tomato. Genes Dev.

[CR15] Nishizawa A, Yabuta Y, Yoshida E, Maruta T, Yoshimura K, Shigeoka S (2006). *Arabidopsis* heat shock transcription factor *A2* as a key regulator in response to several types of environmental stress. Plant J.

[CR16] Nishizawa-Yokoi A, Nosaka R, Hayashi H, Tainaka H, Maruta T (2011). *HsfA1d* and *HsfA1e* involved in the transcriptional regulation of *HsfA2* function as key regulators for the *HSF* signaling network in response to environmental stress. Plant Cell Physiol.

[CR17] Nover L, Bharti K, Döring P, Mishra SK, Ganguli A, Scharf KD (2001) *Arabidopsis* and the heat stress transcription factor world: how many heat stress transcription factors do we need? Cell Stress Chaperones 6:177–89. 10.1379/1466-1268(2001)006<0177:aathst>2.0.co;2 10.1379/1466-1268(2001)006<0177:aathst>2.0.co;2 PMC434399 11599559

[CR18] Ogawa D, Yamaguchi K, Nishiuchi T (2007). High-level overexpression of the *Arabidopsis HsfA2* gene confers not only increased thermotolerance but also salt/osmotic stress tolerance and enhanced callus growth. J Exp Bot.

[CR19] Redondo-Gómez S, Rout GR, Das AB (2013). Abiotic and biotic stress tolerance in plants. Molecular stress physiology of plants.

[CR20] Scharf K-D, Berberich T, Ebersberger I, Nover L (2012). The plant heat stress transcription factor (Hsf) family: structure, function and evolution. Biochim Biophys Acta.

[CR21] Schneider CA, Rasband WS, Eliceiri KW (2012). NIH Image to ImageJ: 25 years of image analysis. Nat Methods.

[CR22] Schöffl F, Prandl R, Reindl A (1998). Update on signal transduction regulation of the heat-shock response. Plant Physiol.

[CR23] Schramm F, Larkindale J, Kiehlmann E, Ganguli A, Englich G, Vierling E, von Koskull-Döring P (2008). A cascade of transcription factor *DREB2A* and heat stress transcription factor *HsfA3* regulates the heat stress response of *Arabidopsis*. Plant J.

[CR24] Shinozaki K, Yamaguchi-Shinozaki K (1996). Molecular responses to drought and cold stress. Curr Opin Biotechnol.

[CR25] Siddique M, Gernhard S, von Koskull-Döring P, Vierling E, Scharf K-D (2008). The plant sHSP superfamily: five new members in *Arabidopsis thaliana* with unexpected properties. Cell Stress Chaperones.

[CR26] Sugio A, Dreos R, Aparicio F, Maule AJ (2009). The cytosolic protein response as a subcomponent of the wider heat shock response in *Arabidopsis*. Plant Cell.

[CR27] Swindell WR, Huebner M, Weber AP (2007). Transcriptional profiling of *Arabidopsis* heat shock proteins and transcription factors reveals extensive overlap between heat and non-heat stress response pathways. BMC Genomics.

[CR28] Yeh C-H, Wu S-J, Tsai Y-F, Chen H-Y, Lin C-Y (2007). Physiological effects of azetidine on cellular leakage in soybean seedlings. Plant Sci.

